# Design and Binding Affinity of Antisense Peptides for Snake Venom Neutralization †

**DOI:** 10.3390/molecules30040903

**Published:** 2025-02-15

**Authors:** Ivan Biruš, Tino Šeba, Marin Marić, Mario Gabričević, Tin Weitner

**Affiliations:** Department of General and Inorganic Chemistry, University of Zagreb Faculty of Pharmacy and Biochemistry, A. Kovačića 1, 10000 Zagreb, Croatia

**Keywords:** snake venom, peptide design, binding affinity, fluorescence spectroscopy, molecular docking

## Abstract

Snakebites are a significant public health problem in many tropical and subtropical regions, causing extensive morbidity and mortality. Traditional snake antivenoms face multiple challenges, including allergenicity, high production costs, and logistical difficulties, highlighting the urgent need for new therapeutic approaches. This pilot study explores the potential of oligopeptides as therapeutic inhibitors targeting the neurotoxic sites of ammodytoxin A (AtxA; PDB: 3G8G) from *Vipera ammodytes*. We selected two sense oligopeptides to represent critical neurotoxic regions of AtxA as targets for inhibition by complementary antisense peptides. Utilizing a heuristic antisense peptide design based on the molecular recognition theory, we modeled two antisense oligopeptides as complementary counterparts for each sense oligopeptide. The modeled sense and antisense peptides were commercially synthesized, and their binding affinities were evaluated using spectrofluorometric titrations. The determined dissociation constants (*K*_D_) were in the range of 1–10 μM for all sense–antisense pairs, revealing relatively strong binding affinities. Confirmation of sense–antisense peptide binding prompted further investigation into their potential binding to the native target protein through global docking simulations using the HPEPDOCK web server. The results highlight the applicability of molecular recognition theory in the development of antisense peptides that could change therapeutic strategies in various biomedical fields. Further studies are needed to investigate the therapeutic efficacy and broader applications of these peptides.

## 1. Introduction

**Figure 1:**
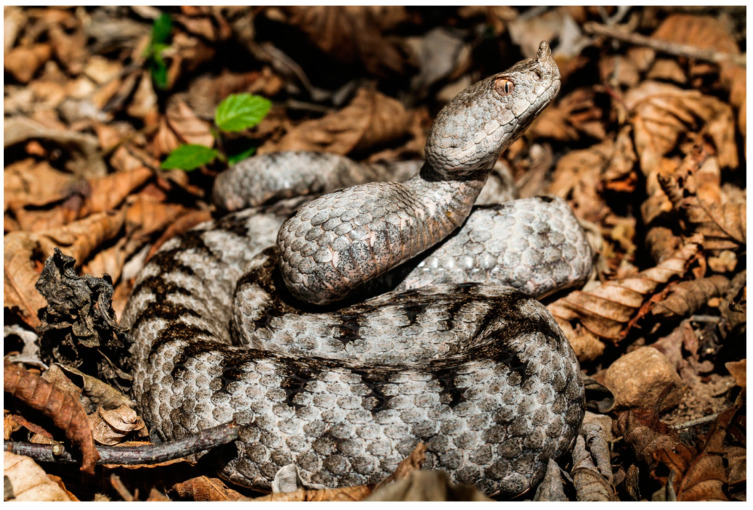
Nose-horned viper (*Vipera ammodytes* (Linnaeus, 1758)) photographed on 26 May 2017, at Belluno, Italy [8].

The nose-horned viper (*Vipera ammodytes*) is a venomous species in the family Viperidae, considered the most dangerous viper in Europe [1]. Males grow up to 100 cm and are typically ash gray, while females, reaching 60 cm, are usually brown, gray-brown, or reddish-brown. Characteristic features include a small ridge on the head and a zigzag pattern along the back (Figure 1). These snakes inhabit dry, sunny, sparsely vegetated areas, particularly karst and rocky regions, and are found across southeastern Europe. In many countries, including Croatia, where the species is strictly protected, it is considered medically significant due to the venom’s potency [2].

Viper bites most commonly occur from July to October, with middle-aged farmers and children being the most frequent victims [3,4]. Local symptoms include edema, pain, skin discoloration, ecchymosis, and regional lymphadenitis. In severe cases, necrosis, thrombophlebitis, and cellulitis may occur. Systemic symptoms often involve gastrointestinal disturbances (nausea, vomiting, diarrhea) and mild hyperthermia (<38 °C). Cardiovascular effects include tachycardia and fainting, while neurological symptoms may involve cranial nerve paralysis, such as ptosis due to oculomotor nerve involvement. In rare cases, central nervous system depression, hypotension, shock, and reversible renal or hepatic dysfunction are observed. Hematological findings typically include leukocytosis, with anemia and thrombocytopenia being less common [4,5,6,7].

### 1.1. Composition of the Vipera ammodytes Venom

The venom of *Vipera ammodytes* exhibits a wide range of pharmacological effects due to its heterogeneous composition, which varies both at the population and individual levels, a phenomenon known as intraspecies variability. For instance, as many as 57 proteins from 16 different families were identified in the venom from different Croatian specimens [9]. These proteins are generally divided into enzymatic and non-enzymatic groups. Enzymatic proteins include serine proteases (SVSPs), metalloproteinases (SVMPs), secretory phospholipases A2 (sPLA2), and L-amino acid oxidases (LAAOs). Non-enzymatic proteins consist of C-type lectin-like proteins (CLPs), cysteine-rich secretory proteins (CRISPs), disintegrins (Dis), nerve growth factors (NGFs), vascular endothelial growth factors (VEGFs), Kunitz-type serine protease inhibitors (SPIs), metalloproteinase inhibitors (MPIs), natriuretic peptides (NPs), and bradykinin-potentiating peptides (BPPs) [9,10,11,12].

Ammodytoxin A (AtxA; PDB: 3G8G) is the most potent neurotoxin in *Vipera ammodytes* venom belonging to the sPLA2 group IIa family [13]. Among the three known ammodytoxins (AtxA, AtxB, AtxC), AtxA demonstrates the highest potency in disrupting neuromuscular function and has been selected as the target for this study due to its significant neurotoxic effects. AtxA acts primarily through presynaptic inhibition of acetylcholine release, resulting in paralysis [14,15]. Its neurotoxic mechanism involves both extracellular enzymatic activity and intracellular interactions with target proteins, though the complete mechanism of its translocation into neurons remains unknown [16,17].

**Figure 2:**
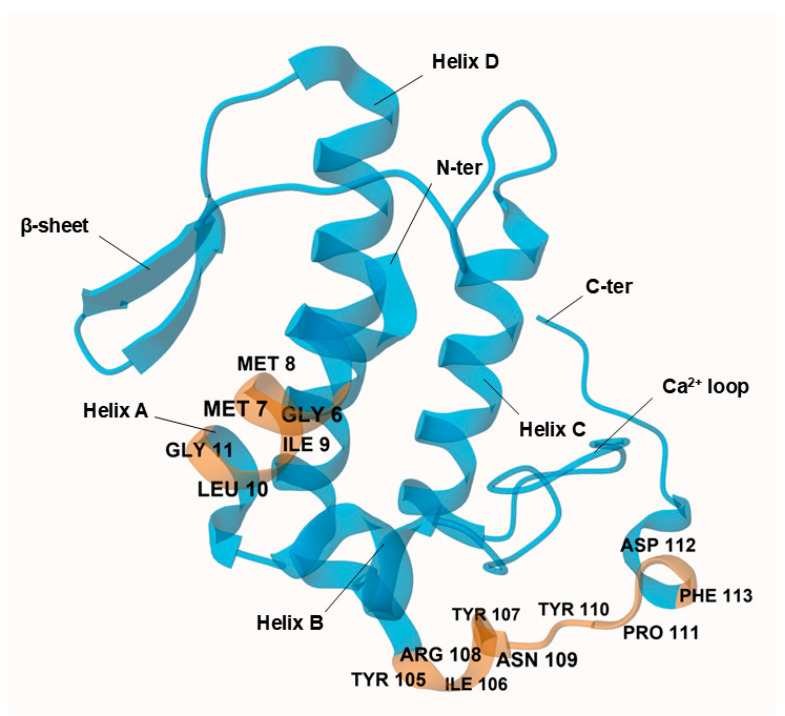
Crystal structure of ammodytoxin A (AtxA; PDB: 3G8G) is shown in blue, with targeted neurotoxic sites corresponding to the modeled sense peptide sequences highlighted in orange. Key residues at these sites are explicitly labeled for clarity. Visualization of the structure was performed using the Mol* Viewer web application [23]. A single polypeptide chain of the crystallized homodimer is shown, with sulfate ions and water molecules omitted from the crystal structure for clarity. Residues are numbered according to the numbering system proposed by Renetseder et al. [24].

Structurally, AtxA comprises 122 amino acids, with seven disulfide bonds and a canonical group IIa sPLA2 architecture: α-helix A at the N-terminus (positions 1–14), short helix B (positions 16–22), a calcium ion-binding loop (positions 25–35), long α-helix C (positions 39–57), a loop before the antiparallel β-sheet (positions 75–58 and 81–84), long α-helix D (positions 89–109), and a C-terminal extension (positions 110–133) with two short helical turns [18] (Figure 2). In its crystallized form, AtxA is a homodimer with two asymmetric subunits linked by hydrophobic interactions between the α-helix A at the N-terminus and hydrogen bonds between Ser23 and Arg72.

AtxA does not have a single neurotoxic site; instead, different parts of the molecule, particularly amino acids at the N- and C-termini, contribute to its neurotoxicity [19]. The AtxA catalytic site is formed by His48, Tyr52, Tyr73, and Asp99, surrounded by the interfacial binding surface formed by Leu2, Leu3, Leu19, Thr20, Phe24, Val31, Ser67, Lys69, Thr70, Arg72, Lys74, Tyr113, Arg118, Asn119, and Phe124 [20]. The side chains of these residues are flexible so that they may allow optimal orientation for phospholipid binding and hydrolysis [18]. In addition, the YIRN cluster (Y115, I116, R118, N119) and residues such as F124 at the C-terminus and F24 at the N-terminus are crucial for neurotoxicity and enzymatic activity [21,22].

### 1.2. Molecular Recognition Theory

Molecular recognition theory states that sense peptides and their complementary antisense peptides interact based on their contrasting hydropathic properties [25,26,27]. The complementary secondary and tertiary structures of the sense and antisense peptides enable specific interactions with high affinity [28]. In addition to hydrophobic interactions, electrostatic forces and hydrogen bonding contribute to the stability of the sense–antisense complex [29].

Binding between sense and antisense peptides is conformationally degenerate, occurring at multiple sites with clusters of non-covalent bonds. Longer peptides tend to exhibit higher binding affinity [30,31]. Due to the degeneracy of the genetic code, most amino acids can interact with multiple others, allowing several possible complementary antisense peptides for a given sense peptide (Table 1) [25,26]. Reading codons in the 3′-5′ direction can reduce this degeneracy, simplifying antisense peptide design [32].

Although a clear algorithm for antisense peptide design remains elusive, peptides can be designed based on the sense peptide’s primary structure, with amino acids selected to complement those in the sense peptide [26]. Antisense peptides can inhibit sense peptides by forming a complex, reducing the concentration of free sense peptide, acting as partial receptor antagonists, or via mechanisms that are not yet fully understood [25].

### 1.3. Scope and Justification

Snake antivenom immunoglobulins are currently the only therapeutic option for treating envenomations. However, traditional antivenoms have significant limitations, including allergenicity, limited shelf life, high production and distribution costs, and the need for cold storage, which poses logistical challenges, particularly in rural areas. Furthermore, variability in the quality and efficacy of antivenoms can lead to unpredictable treatment outcomes. Given these drawbacks and the global burden of snakebites, there is an urgent need for alternative therapeutics to address this serious health issue [33].

This pilot study aims to experimentally determine the binding affinity of antisense peptides to sense peptides representing neurotoxic sites in AtxA. By inhibiting these critical neurotoxic sites, antisense peptides may block the biological activity of the toxin, potentially serving as a more effective treatment for envenomation. Antisense peptides offer several advantages over immunoglobulin-based antivenoms, including reduced immunogenicity, lower production costs, and increased stability, which simplifies storage and transport. The antisense peptides were designed based on the molecular recognition theory. For a peptide length of *n* amino acids, this design significantly improves the efficiency of peptide library screening by reducing the solution space by a factor of (20/1.35)^n^ compared to the random peptide library approach [34].

The main objective of this work was to provide a proof-of-concept for the sense–antisense peptide binding approach, focusing on fluorescence spectroscopy as a convenient, low-cost, and high-throughput method for assessing peptide interactions. Fluorescence spectroscopy was particularly suitable for this initial investigation due to the intrinsic fluorescence of the tyrosine residues and allowed straightforward monitoring of binding events.

To further explore the potential of antisense peptides as therapeutic agents, docking simulations were conducted to evaluate their interaction with the native AtxA structure. These simulations provide insight into the binding mechanism and complement the experimental findings by offering a detailed molecular perspective on the peptide–toxin interaction.

## 2. Results and Discussion

### 2.1. Physico-Chemical Properties of the Sense and Antisense Peptides

**Figure 3:**
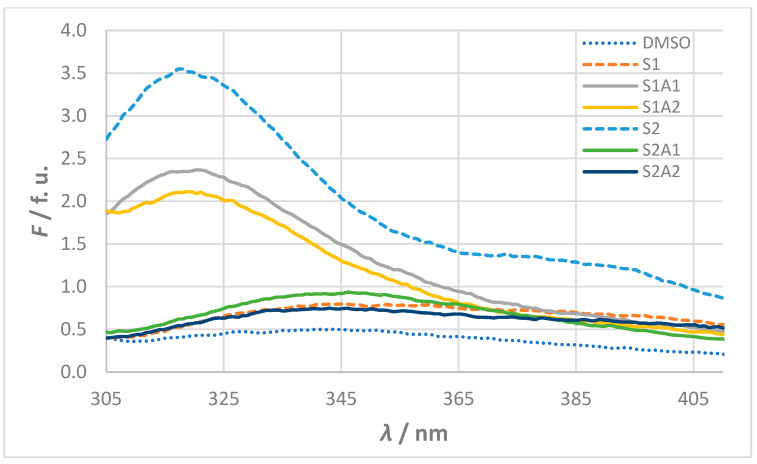
Fluorescence spectra for stock solutions of sense and antisense peptides (*c* = 10^−5^ mol L^−1^). Fluorescence (*F*) is given in fluorescence units (f. u.) calculated as the ratio of signals from the sample and reference photomultiplication tube. The spectra of the sense peptides S1 and S2 are shown with dashed lines, while the spectra of the antisense peptides (S1A1, S1A2, S2A1, and S2A2) are shown with solid lines. The baseline DMSO solvent spectrum shown as a dotted line serves as a reference.

The fluorescence spectra of the 10^−5^ mol L^−1^ solutions of sense and antisense peptides in 100 % DMSO are shown in Figure 3. The measured intensities and wavelengths of the fluorescence maxima (*F* and *λ*_max_) are summarized in Table 2, together with the computed values for theoretical p*I*, estimated half-life, instability index (II), aliphatic index (AI), and grand average of hydropathicity (GRAVY) obtained from the ExPASy ProtParam server.

The fluorescence behavior observed in the experimental assays correlates with the peptide properties computed in silico. High fluorescence intensities for S2, S1A1, and S1A2 (*F* > 2 f. u.) are associated with negative GRAVY values, indicating their hydrophilic nature. Conversely, the low fluorescence intensities of S1, S2A1, and S2A2 (*F* < 1 f. u.) align with their higher aliphatic indices, which suggest less hydrophilic structures. These opposite hydropathic properties for sense and antisense peptides are consistent with peptide design based on the molecular recognition theory.

The p*I* values further highlight differences that could influence binding interactions. For S1 (p*I* = 5.52), the slightly higher p*I* values of S1A1 (p*I* = 5.93) and S1A2 (p*I* = 6.66) indicate potential electrostatic interactions. A more pronounced difference is seen between S2 (p*I* = 5.83) and its antisense peptides S2A1 and S2A2 (both p*I* ≈ 8.7). These p*I* variations may play a critical role in the binding efficiency and stability of sense and antisense peptides under different pH conditions.

The high values of II for SHHYEP and SHHYSP (II > 100, Table 2) indicate that they may be susceptible to rapid degradation or structural instability under in vitro conditions, especially in the absence of stabilizing factors such as folding into secondary or tertiary structures. Guruprasad et al. found that certain amino acids such as serine (S) and proline (P) contribute significantly to instability when present in high proportions [36]. Serine, with its polar hydroxyl group, often disrupts hydrophobic interactions, while the rigid cyclic structure of proline is known to break secondary structure elements, which can contribute to the degradation of short peptides such as SHHYEP and SHHYSP. This is also consistent with the predicted values of AI = 0 for these two peptides, indicating the absence of stabilizing aliphatic residues, which may affect their robustness under physiological or storage conditions [37].

The value of II < 40 for the corresponding sense peptide GMMILG indicates a more stable structure compared to SHHYEP and SHHYSP. This is likely due to the presence of methionine (M) and leucine (L), which are aliphatic residues that tend to enhance hydrophobic interactions and maintain the structural integrity of small peptides. Again, the high value of AI = 130 for GMMILG probably confirms its resistance to degradation. To improve the stability of SHHYEP and SHHYSP, modifications such as N-terminal acetylation or C-terminal amidation could be considered [38]. In vivo stability may differ from in vitro predictions, so these high instability values warrant further investigation, especially if these peptides are being considered for therapeutic applications.

### 2.2. Fluorescence Assay of the Interaction Between Sense and Antisense Peptides

The S1 peptide lacks aromatic residues and shows no fluorescence, whereas both S1A1 and S1A2 contain tyrosine, which elicits fluorescence. In this case, S1 was titrated into antisense peptide solutions (S1A1 and S2A2). Conversely, the S2 peptide contains several tyrosines and shows fluorescence, whereas S2A1 and S2A2 do not. Therefore, the antisense peptides (S2A1 and S2A2) were titrated into a solution of S2. Titration points were selected to ensure a range of guest-to-host concentration ratios, starting with a guest concentration lower than the host, approximately equal, and then in excess [39].

For all titrations, a 5 × 10^−6^ mol L^−1^ solution of the fluorescent host (either S1A1, S1A2, or S2) was titrated with a solution of the non-fluorescent binding guest (either S1, S2A1, or S2A2) at a concentration ratio [guest]/[host] ranging from ≈1:2.5 to ≈100:1. It is important to note that the fluorescence intensity may not increase linearly at higher analyte concentrations due to the inner filter effect [40]. Therefore, in all working solutions, the absorbance was kept below 0.05 at both the excitation and emission wavelengths (typically *A*_280_ ≈ 0.02).

**Figure 4:**
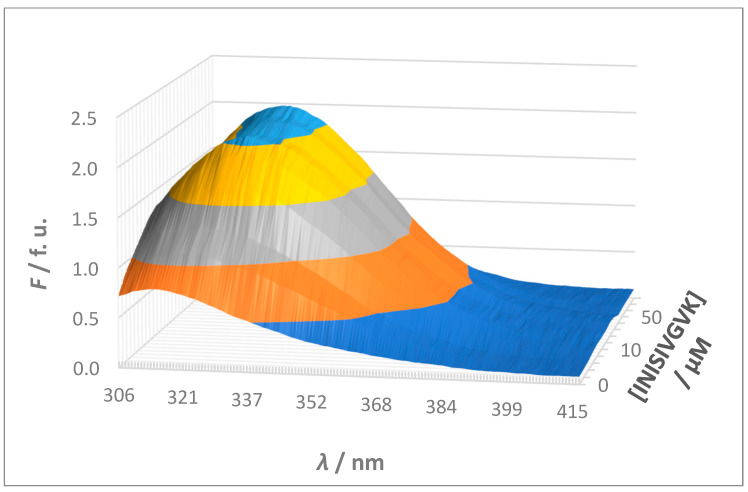
A typical spectrofluorimetric titration illustrating the interaction between sense and antisense peptides shown as a 3-dimensional diagram: a 5 × 10^−6^ mol L^−1^ solution of the fluorescent host (S2, YIYRNYPDF) was titrated with a solution of the non-fluorescent binding guest (S2A1, INISIVGVK) at a concentration ratio [guest]/[host] ranging from ≈1:2.5 to ≈100:1 (i.e., ≈0.4 to ≈100). Different colors represent fluorescence intensity (*F*) levels along the y-axis, aiding in visual interpretation of the data. The colors correspond to fluorescence units (f. u.) in increments of 0.5 starting from zero: dark blue (lowest intensity), orange, gray, yellow, and light blue (highest intensity).

A typical spectrofluorimetric titration illustrating the interaction between sense and antisense peptides is shown in Figure 4. In all titrations, the increasing concentration of the non-fluorescent guest resulted in an increased fluorescence intensity, accompanied by a red shift in the fluorescence emission maximum. This combined effect strongly confirms the formation of a complex between sense and antisense peptides caused by changes in the local environment of the fluorophore upon binding.

The observed red shift in fluorescence emission suggests that the tyrosine (Y) fluorophore is buried in the binding interface of sense and antisense peptides and is thus shielded from the polar solvent. This hydrophobic environment probably stabilizes the excited state of the fluorophore and causes the shift to longer wavelengths. Furthermore, the increase in fluorescence intensity indicates an increase in the quantum yield of the fluorophore. This increase is likely due to the fluorophore becoming more rigid or less solvent-exposed after binding, thereby reducing non-radiative decay pathways and enhancing fluorescence. While it is known that a red shift can also occur when a fluorophore becomes more exposed to a polar solvent, such an event is usually accompanied by a decrease in fluorescence intensity due to solvent relaxation effects. In this study, the simultaneous increase in fluorescence intensity and red shift indicates that the fluorophore is not more exposed to the solvent, but rather shielded in a hydrophobic binding environment [41,42].

### 2.3. Binding Affinity of Sense and Antisense Peptides

The results of global analysis of the 3D surfaces obtained by titration of sense and antisense peptides using the SPECFIT software were obtained as logarithms of association constants (log *K*_A_). The corresponding values of dissociation constants calculated as $K_{D}=10^{-\log K_{A}}$ and the corresponding standard deviations calculated as $\sigma (K_{D})=\left|K_{D}\right|\left|-\ln\left(10\right)\sigma (\log K_{A})\right|$ are listed in Table 3. In general, all obtained *K*_D_ values were within the typical range observed for peptide–protein interactions, which are generally in the micromolar to low nanomolar range and suggest moderate to strong binding affinity [43]. As expected, the obtained *K*_D_ values are also higher than those usually seen in antibody–antigen interactions, which typically range from low nanomolar to picomolar [44].

The lower *K*_D_ value for the S1A2 interaction (*K*_D_ = 1.4 μM) compared to S1A1 (*K*_D_ = 4.9 μM) suggests that serine (S) at position 5 in S1A2 enhances the interaction with S1 more effectively than glutamate (E) at the corresponding position in S1A1, resulting in increased binding affinity for S1A2. At physiological pH, serine contains a polar but uncharged hydroxyl group in its aliphatic side chain, which facilitates hydrogen bonding and contributes to binding specificity and affinity. It is a common residue in the complementarity-determining regions (CDRs) of antibodies, where it participates in antigen recognition. On the other hand, glutamate is negatively charged at physiological pH, with a terminal carboxyl group that supports ionic interactions and contributes to solubility and stability, but its larger, charged side chain may introduce steric hindrance or electrostatic repulsion in certain binding contexts [45].

The dissociation constant for S2A1 (*K*_D_ = 1.1 μM) is lower compared to S2A2 (*K*_D_ = 8.9 μM), suggesting that isoleucine (I) at positions 1 and 5, and valine at position 8 in S2A1 contribute more favorably to the interaction than the valine (V) at positions 1 and 5 and isoleucine at position 8 in S2A2. In general, valine is less commonly found at antigen-binding interfaces, whereas isoleucine is among the top residues involved in antigen–antibody interactions [45]. The specific positioning of these hydrophobic residues may influence how well the peptides align, potentially forming more favorable interactions through hydrophobic contact surfaces, thereby enhancing the overall binding affinity. These findings highlight how subtle differences in residue composition and positioning can dramatically impact peptide–peptide interactions, emphasizing the need for precise sequence design in optimizing binding affinity.

In all titrations, spectral analysis using singular value decomposition (SVD) in SPECFIT software identified at least two spectrally significant species, corresponding to the fluorescent host and the host–guest complex, accounting for over 94% of the observed spectral variation across all wavelengths and concentrations (Figure S1, Supporting Information) [46]. This suggests a 1:1 interaction ratio between sense and antisense peptides, consistent with molecular recognition theory. Given the relatively small size of both sense peptides and their antisense counterparts (six or nine amino acids), simultaneous binding of multiple antisense molecules to a single sense peptide is thermodynamically unlikely [39].

**Figure 5:**
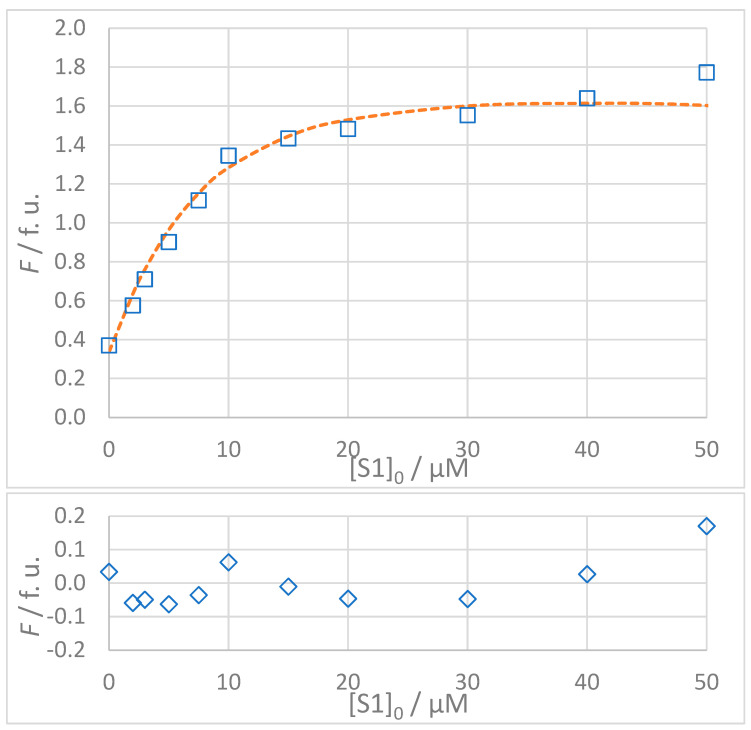
(**Top**): Dependence of observed (*F*_obs_, **□**) and calculated (*F*_calc_, **---**) fluorescence on the concentration of GMMILG (S1) peptide: [S1A1]_0_ = 5 × 10^−6^ mol L^−1^, *t* = 25 °C, 100% DMSO. The values of *F*_calc_ were calculated according to the 1:1 binding model using SPECFIT software (*K*_D_ = 4.9 μM). (**Bottom**): Residuals of fit at 330 nm (**◊**) calculated as *F*_obs_ − *F*_calc_.

**Figure 6:**
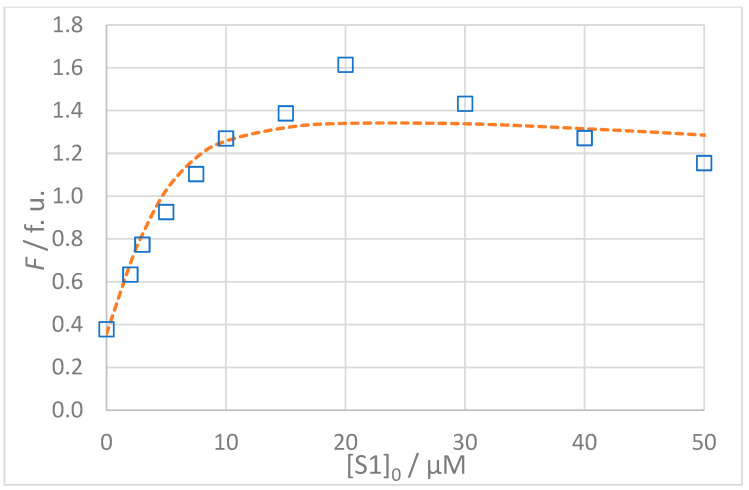

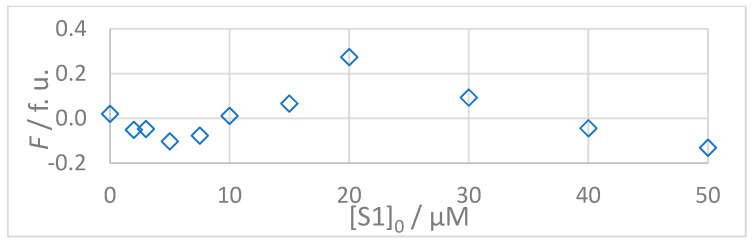
(**Top**): Dependence of observed (*F*_obs_, **□**) and calculated (*F*_calc_, **---**) fluorescence on the concentration of GMMILG (S1) peptide: [S1A2]_0_ = 5 × 10^−6^ mol L^−1^, *t* = 25 °C, 100% DMSO. The values of *F*_calc_ were calculated according to the 1:1 binding model using SPECFIT software (*K*_D_ = 1.4 μM). (**Bottom**): Residuals of fit at 330 nm (**◊**) calculated as *F*_obs_ − *F*_calc_. See text for a discussion of the impact of a potential outlier at [S1]_0_ = 20 μM.

**Figure 7:**
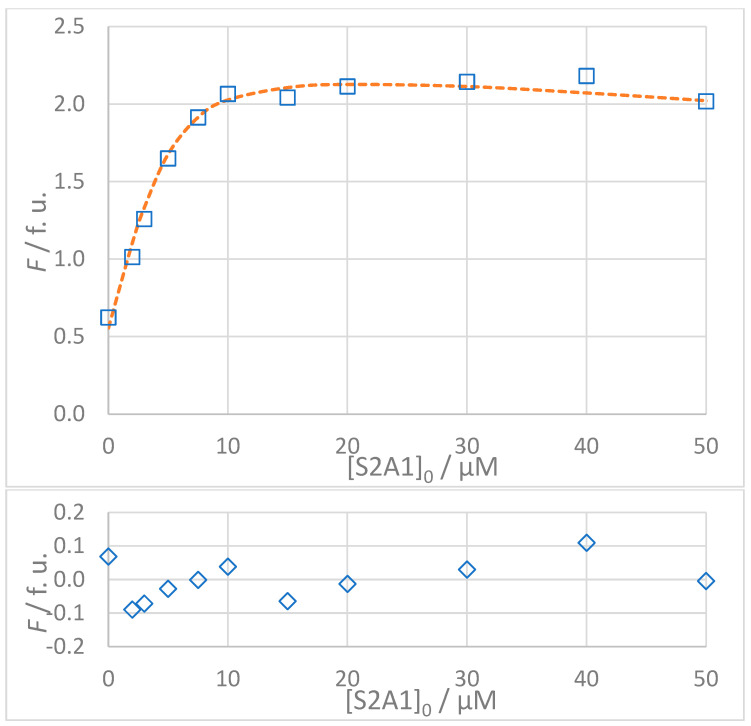
(**Top**): Dependence of observed (*F*_obs_, **□**) and calculated (*F*_calc_, **---**) fluorescence on the concentration of INISIVGVK (S2A1) peptide: [S2]_0_ = 5 × 10^−6^ mol L^−1^, *t* = 25 °C, 100% DMSO. The values of *F*_calc_ were calculated according to the 1:1 binding model using SPECFIT software (*K*_D_ = 1.1 μM). (**Bottom**): Residuals of fit at 330 nm (**◊**) calculated as *F*_obs_ − *F*_calc_.

**Figure 8:**
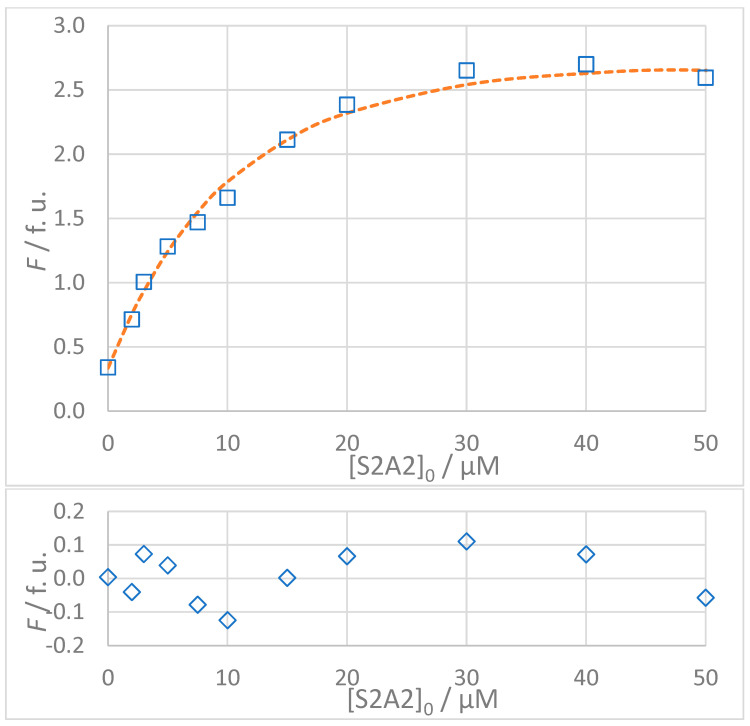
(**Top**): Dependence of observed (*F*_obs_, **□**) and calculated (*F*_calc_, **---**) fluorescence on the concentration of VNISVIGIK (S2A2) peptide: [S2]_0_ = 5 × 10^−6^ mol L^−1^, *t* = 25 °C, 100% DMSO. The values of *F*_calc_ were calculated according to the 1:1 binding model using SPECFIT software (*K*_D_ = 8.9 μM). (**Bottom**): Residuals of fit at 330 nm (**◊**) calculated as *F*_obs_ − *F*_calc_.

The titration data for all interactions listed in Table 3 are shown in Figure 5, Figure 6, Figure 7 and Figure 8. The top panel in each figure shows the dependence of the observed fluorescence intensity (*F*_obs_) on the concentration of the non-fluorescent guest together with the calculated fluorescence values (*F*_calc_) which were determined using the SPECFIT software. The good agreement between *F*_obs_ and *F*_calc_ at 330 nm, close to the fluorescence maximum of the host–guest complex, is shown by the close overlap of the data points and the fitted curve. The residuals of the fit, shown in the bottom panels of the figures, are typically small and randomly distributed around zero, indicating a reliable fit with no systematic deviations. The only notable exception is the S1A2 titration (Figure 6) with a probable outlier point at 20 μM. An attempted fit using a more complex model including both 1:1 and 1:2 binding was unsuccessful. Furthermore, removing the outlier point resulted in only a negligible change in the calculated dissociation constant (*K*_D_ = 1.4 (0.4) μM vs. *K*_D_ = 1.4 (0.5) μM). This outcome highlights the robustness of the global spectral analysis supported by SVD in SPECFIT software and confirms the applicability of the 1:1 binding model to the studied sense and antisense peptides.

### 2.4. Molecular Docking of the Sense and Antisense Peptides

Docking simulations were performed using the HPEPDOCK web server to evaluate the interactions between the sense peptides (S1, S2) and the corresponding antisense peptides (S1A1, S1A2, S2A1, S2A2). The first two top-scoring models for S1A1 and the top-scoring model for S1A2 failed to generate valid 3D structures and were excluded from further analysis. The valid docking scores ranged from −85.504 to −75.083 for S1A1 and −86.267 to −76.752 for S1A2, while S2A1 and S2A2 had ranges from −108.091 to −96.305 and −112.690 to −94.820, respectively.

Analysis of the major interactions in ChimeraX [47] reveals distinct binding features between the S1 and S2 sense regions and differences between antisense variants A1 and A2 (Figure S3 and Table S1, Supporting Information). All peptide pairs are dominated by hydrophobic interactions, with additional contributions from potential hydrogen bonding, sulfur-π and electrostatic interactions. In the S1 sense region, both antisense peptides (S1A1 and S1A2) exhibited strong hydrophobic interactions involving methionine (M) residues. An additional Ile-Tyr interaction could explain the stronger experimental binding affinity of S1A2 (*K*_D_ = 1.4 μM) compared to S1A1 (*K*_D_ = 4.9 μM).

In the S2 sense region, peptide pairs were also dominated by hydrophobic interactions, especially those involving tyrosine (Y). However, the experimental binding affinity data revealed a significant difference between these two antisense peptides, with S2A1 showing strong binding (*K*_D_ = 1.1 μM), while S2A2 exhibited a much weaker affinity (*K*_D_ = 8.9 μM). This discrepancy suggests that the specific interaction network in S2A1, especially the involvement of Tyr-Ile, may provide superior stabilization compared to the interactions observed in S2A2.

### 2.5. Molecular Docking of the Antisense Peptides and Ammodytoxin A

**Figure 9:**
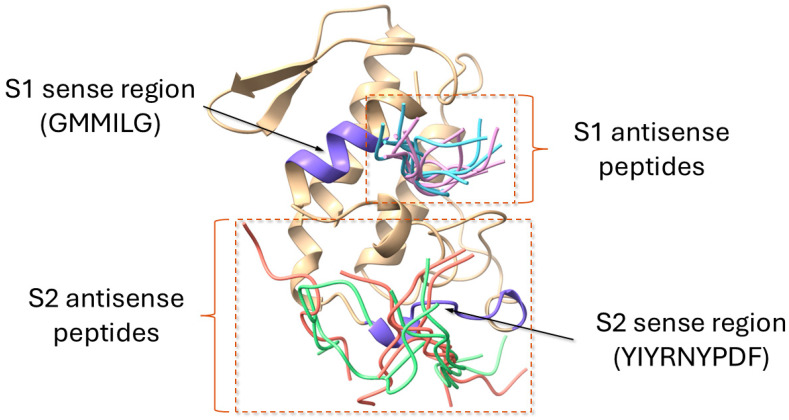
Summary of the global docking simulations of ammodytoxin A (AtxA; PDB: 3G8G) with modeled antisense peptides obtained using the HPEPDOCK web server [48]. The AtxA structure is displayed as a cartoon representation in yellow, with the sense regions S1 and S2 highlighted in purple. Sequences of the antisense peptides are listed in Section 3. The top five docking models for each antisense peptide are shown as tube representations: S1A1 (light blue), S1A2 (pink), S2A1 (green), and S2A2 (red). The variability in docking orientations reflects the flexibility of the peptide binding to AtxA. Visualization of structures was performed using the USCF ChimeraX software (version 1.8) [47].

The docking simulations for antisense peptides S1A1, S1A2, S2A1, and S2A2 onto AtxA provided further insights into their binding orientations and predicted affinities. The docking results highlight the spatial localization of the antisense peptides near the corresponding sense regions, with S1A1 and S1A2 docking near the S1 region and S2A1 and S2A2 docking near the S2 region. For each peptide, the 10 top-ranking docking models obtained from the HPEPDOCK web server were analyzed, with overlays highlighting the variability in binding configurations (for clarity, only top five models for each peptide are shown in Figure 9). The top-ranked models were further examined to identify the most favorable interaction sites.

**Figure 10:**
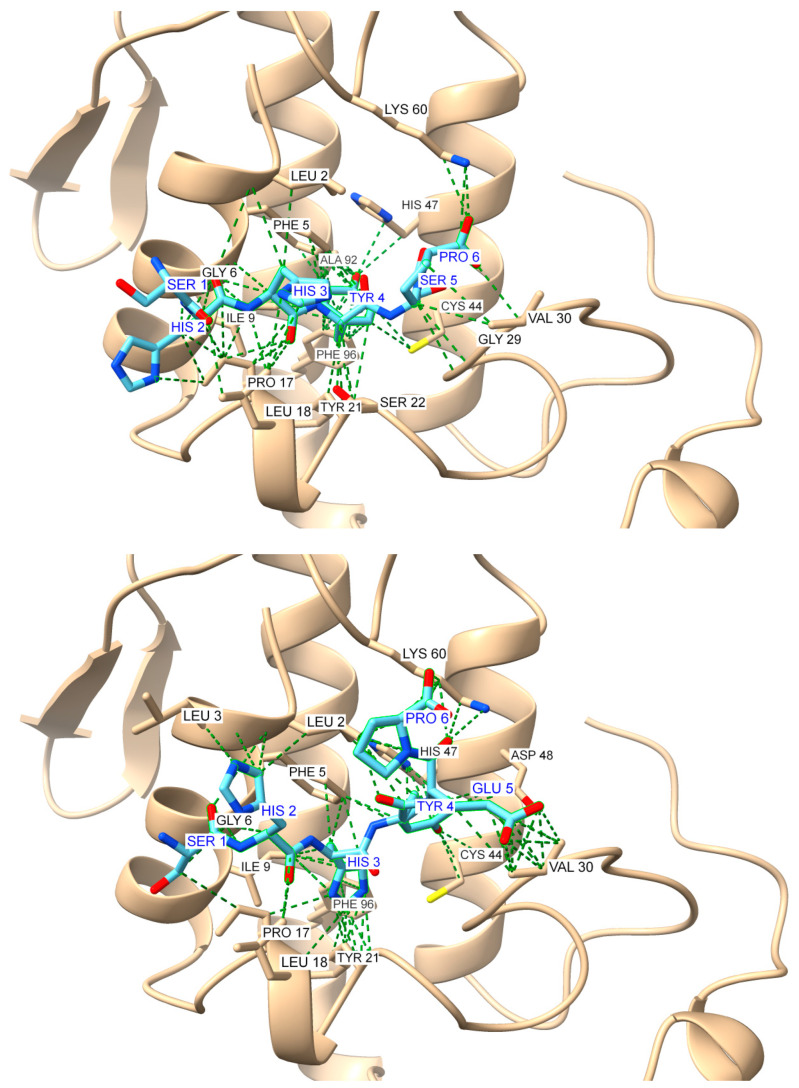
Global docking simulations of ammodytoxin A (AtxA; PDB: 3G8G, tan cartoon) S1 region represented by GMMILG sense peptide with modeled antisense peptides: (**Top**): top-scoring model for the GMMILG-SHHYEP (S1A1) complex (score: −184.958); (**Bottom**): top-scoring model for the GMMILG-SHHYSP (S1A2) complex (score: −188.883). Results were obtained using the HPEPDOCK web server [48], while the visualization of the structures and the contact analysis were performed using the USCF ChimeraX software (version 1.8) [47]. Interactions are highlighted as green dashed pseudobonds, with atom colors distinguishing key elements: oxygen (red), nitrogen (blue), and sulfur (yellow). Residues involved in interactions are labeled in black for AtxA and blue for the antisense peptides.

The overlay of the top five docking models for S1A1 revealed rather uniform binding orientations on the AtxA surface, suggesting limited flexibility in the binding interface. The docking scores ranged from −184.96 for the top model to −162.13 for the fifth-ranked model, indicating relatively high binding affinity. Similarly to S1A1, the docking results for S1A2 displayed consistent overlaps near key regions of the AtxA structure. The top docking model score of -188.88 suggests slightly stronger binding compared to S1A1. The score for the fifth-ranked model was −170.67, confirming a strong overall binding profile. Representative models for S1A1 and S1A2 peptides are shown in Figure 10.

**Figure 11:**
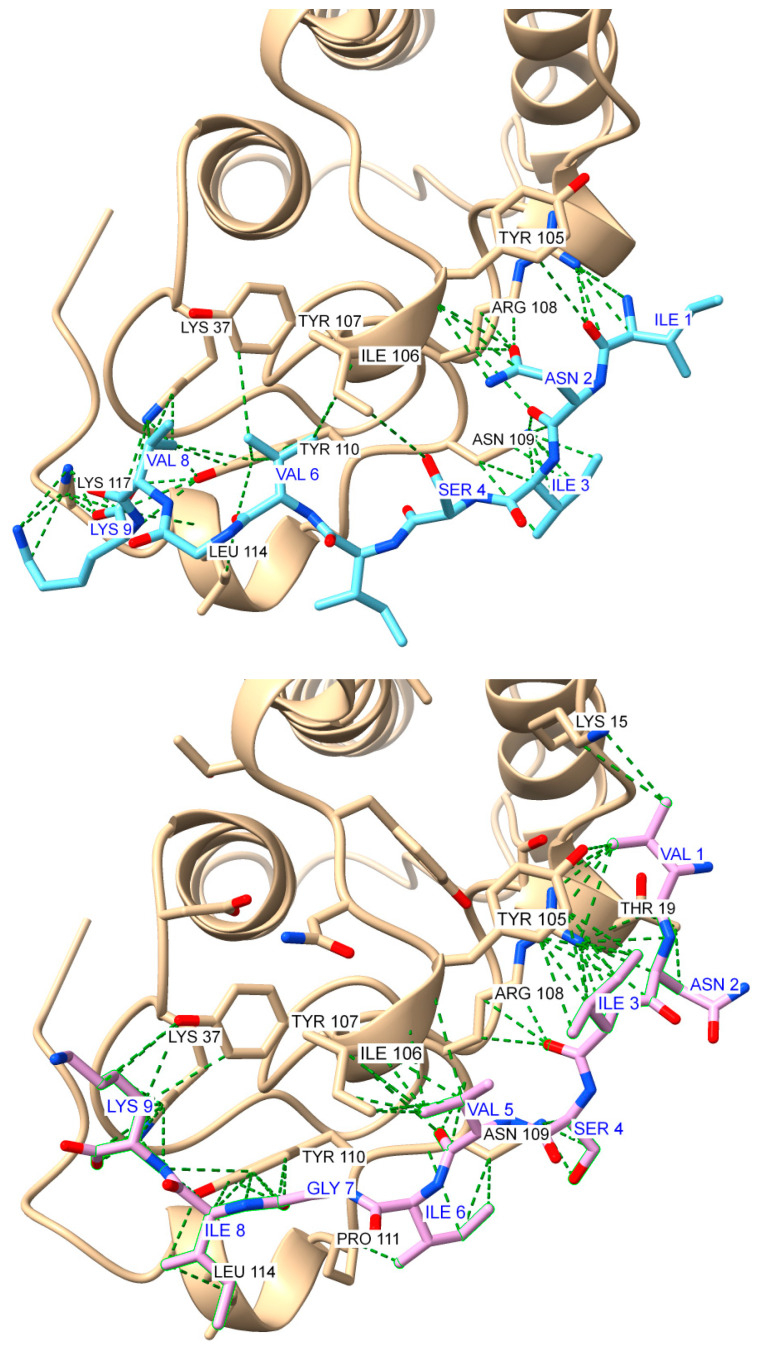
Global docking simulations of ammodytoxin A (AtxA; PDB: 3G8G, tan cartoon) S2 region represented by YIYRNYPDF sense peptide with modeled antisense peptides: (**Top**): top-scoring model for the YIYRNYPDF-INISIVGVK (S2A1) complex (score: −122.048); (**Bottom**): second top-scoring model for the YIYRNYPDF-VNISVIGIK (S2A2) complex (score: −122.081). Results were obtained using the HPEPDOCK web server [48], while the visualization of the structures and the contact analysis were performed using the USCF ChimeraX software (version 1.8) [47]. Interactions are highlighted as green dashed pseudobonds, with atom colors distinguishing key elements: oxygen (red), nitrogen (blue), and sulfur (yellow). Residues involved in interactions are labeled in black for AtxA and blue for the antisense peptides.

The top five docking models for S2A1 showed more variability in binding configurations compared to S1A1 and S1A2, with a less defined interaction site on AtxA. The docking scores were also slightly weaker, with the top-ranked model scoring −122.05 and the fifth-ranked model scoring −115.37, suggesting lower binding affinity than S1A1 and S1A2. The results for S2A2 showed an even less localized binding region, with the top model appearing slightly misaligned relative to the S2 sequence in AtxA. The second-ranked model aligned more closely with the expected binding region and was therefore selected as representative. The top docking score for S2A2 was −123.50, with the second model scoring −122.08, suggesting comparable affinities for the two top solutions. The fifth-ranked model scored −113.62. Representative models for S2A1 and S2A2 peptides are shown in Figure 11.

Overall, the docking results indicate that the antisense peptides S1A1 and S1A2 have stronger predicted binding to AtxA, with lower docking scores and lower variability in potential binding sites. This could indicate more specific interactions and potentially increase their ability to inhibit key functional regions of AtxA. In contrast, S2A1 and S2A2 showed less localized binding and slightly weaker affinities, which may suggest less effective interaction or specificity for alternative binding sites on AtxA. These results provide a basis for selecting S1A1 and S1A2 as lead candidates for further studies aimed at inhibiting AtxA activity.

It is important to note that the docking scores are not absolute measures of binding affinity. They are influenced by various factors, including the specific characteristics of each peptide and the conformational flexibility of both the peptide and the protein. As a result, direct comparison of docking scores across different peptide–protein interactions, such as those between AtxA and the antisense peptides S1A1, S1A2, S2A1, and S2A2, may not yield accurate insights into their relative binding strengths.

The experimental binding affinities of sense and antisense peptides, serving as surrogates for AtxA neurotoxic sites (Table 3), generally align with the docking predictions for the S1 interactions, where both approaches identify S1A2 as the stronger binder (*K*_D_ = 1.4 μM). This consistency underscores the utility of docking simulations for providing initial insights into binding interactions.

However, for S2 interactions, the docking results did not accurately reflect the significant difference in binding affinity observed experimentally between S2A1 and S2A2. The experimental *K*_D_ values were 1.1 μM and 8.9 μM, respectively, showing a clear difference in binding strength. In contrast, the docking scores for these interactions were −122.05 for S2A1 and −123.50 for S2A2, indicating minimal differentiation between the two peptides in simulations. Of note, the top-scoring docking model for S2A2 was misaligned relative to the S2 sequence in AtxA, which could explain its weaker experimental binding. This discrepancy was also observed in the docking results for the sense and antisense peptides (Section 2.4) and highlights the limitations of docking simulations, which may overlook critical contributions of peptide flexibility, solvation effects, or specific molecular interactions that occur in the experimental system.

The results of a detailed contacts analysis in ChimeraX show that the antisense peptides form distinct interactions with AtxA that combine hydrophobic interactions, aromatic stacking, and hydrogen bonding (Table S2, Supporting Information). S2A1 and S2A2 are characterized by hydrogen bonds, in addition to hydrophobic interactions, highlighting their specificity and potential efficacy in binding. In contrast, S1A1 and S1A2 rely more heavily on hydrophobic and aromatic stacking for their stabilization profiles.

Comparative analysis of docking interactions and molecular recognition pairings revealed an overlap between computational predictions and theoretical sense–antisense pairings listed in Table 1. Several key interactions, such as Cys-Tyr, Ile-Tyr, and Tyr-Val, agree with established molecular recognition pairings, confirming complementarity between these residues. Other interactions, while not directly matching the theoretical pairs, provided additional insight into possible alternative binding modes and unrecognized complementary patterns (Table S2, Supporting Information).

## 3. Materials and Methods

### 3.1. Materials

Six peptides were used in this study: two sense peptides (S1 and S2) and four antisense peptides (S1A1, S1A2, S2A1, and S2A2). The peptides used in this study were purchased from GenScript Biotech B.V. (Rijswijk, The Netherlands; lot no. U648UEK070-1) and the quality was assessed by MS and HPLC (specifications are listed in Table 4). Peptide stock solutions were prepared by dissolving each peptide in 100% dimethyl sulfoxide (DMSO, cat. no. 0453201, Kemika d.d., Zagreb, Croatia) at a concentration of 10^−4^ mol L^−1^ and stored at 4 °C to maintain stability. DMSO was chosen as the solvent due to its low fluorescence intensity and good peptide solubility. Solubility tests showed that the peptides were not completely soluble in water, ethanol, methanol, and isopropanol, but dissolved in DMSO, acetonitrile, and DMF. Acetonitrile and DMF exhibited high fluorescence interference, making DMSO the optimal choice for spectrofluorimetric measurements. The water used in the solubility tests was double-distilled in an all-glass apparatus, while all other solvents were of analytical or HPLC grade, obtained from Kemika d.d., Zagreb, Croatia.

DMSO is a widely used solvent in peptide and protein studies because of its ability to solubilize a wide range of biomolecules. However, its effects on peptide structure and interactions need to be critically considered. Studies have shown that DMSO indirectly affects the hydrogen bonding network and hydration shell of peptides by interacting with the surrounding water molecules rather than forming direct bonds with the peptides themselves [49]. In a fully aqueous system, peptides often rely on water-mediated hydrogen bonds to fold and interact properly. In 100% DMSO, these bonds can be disrupted or replaced by DMSO-mediated effects, potentially altering native-like binding conformations. In addition, DMSO can modulate hydrophobic interactions and either stabilize or destabilize aggregation-prone sequences, depending on the composition of the peptide [50].

Importantly, it has been reported that increasing the concentration of DMSO reduces the number of hydration waters regardless of the amino acid composition. DMSO also uniformly stabilizes different peptide conformations by increasing the kinetic barriers in the conformational energy landscape [51]. This relatively uniform interaction of DMSO with different amino acids suggests that peptides in a DMSO-rich environment can adopt conformations that are less influenced by the specific side-chain properties of individual amino acids. This uniformity can lead to a stabilization of peptide structures that would not occur under aqueous, physiological conditions, where water molecules interact differently with amino acid residues depending on polarity and charge. Consequently, experimental observations made in high concentrations of DMSO may not accurately reflect peptide behavior in vivo, where the solvent environment is predominantly water. Therefore, caution should be exercised when extrapolating data from DMSO-based studies to physiological contexts, as the solvent-specific interactions in DMSO may not correspond to those in natural biological systems.

### 3.2. Antisense Peptide Design

The residues M7, G11, and P24 at the N-terminus and Y115, I116, R118, N119, and P124 at the C-terminus were identified as neurotoxic sites in the structure of AtxA. Therefore, in this study, the AtxA segments (S1 and S2 peptides) were used as surrogates for the neurotoxic sites instead of the AtxA protein in measurements of the binding affinities. This approach allows for the characterization of binding affinities in a simplified system, facilitating the direct measurement of peptide interactions. The primary structure of the S1 peptide is GMMILG (Gly-Met-Met-Ile-Leu-Gly) and corresponds to the amino acid sequence of AtxA from position 6 to 11. The primary structure of the S2 peptide is YIYRNYPDF (Tyr-Ile-Tyr-Arg-Asn-Tyr-Pro-Asp-Phe) and corresponds to the amino acid sequence of AtxA from position 115 to 124.

Based on the structures of peptides S1 and S2, complementary antisense peptides were designed with the highest theoretical binding probability. When designing sense and antisense peptides based on molecular recognition theory, an additional constraint was applied to facilitate the fluorometric binding assay: either the sense or antisense peptide must contain an aromatic amino acid to ensure only one of the peptides is fluorescent, preventing signal overlap and enabling clear detection of binding by fluorescence spectroscopy. For S1, two antisense peptides, SHHYEP (Ser-His-His-Tyr-Glu-Pro, S1A1) and SHHYSP (Ser-His-His-Tyr-Ser-Pro, S1A2), were designed. For S2, another two antisense peptides, INISIVGVK (Ile-Asn-Ile-Ser-Ile-Val-Gly-Val-Lys, S2A1) and VNISVIGIK (Val-Asn-Ile-Ser-Val-Ile-Gly-Ile-Lys, S2A2), were chosen, based on amino acid complementarity (Table 1) [25].

The physical and chemical properties of the sense and antisense peptides were analyzed in silico using the ExPASy ProtParam server (https://web.expasy.org/protparam/ (accessed on 12 June 2024)) [52]. ProtParam is a computational tool that analyzes various physico-chemical properties of proteins based on their sequence. The tool calculates several parameters, including molecular weight, theoretical isoelectric point (p*I*), amino acid and atomic composition, extinction coefficient, estimated half-life, instability index, aliphatic index, and the grand average of hydropathicity (GRAVY).

### 3.3. Fluorescence Assay for the Binding of Sense and Antisense Peptides

Fluorescence spectroscopy was used to monitor the binding interactions between sense and antisense peptides. The changes in fluorescence intensity were measured to quantify the binding affinities (expressed as *K*_D_ values), as the fluorescence signal depends on changes in the fluorophore environment [53,54]. It is known that a peptide exhibits fluorescence when it contains aromatic amino acids (phenylalanine, tyrosine, tryptophan) [55]. Therefore, in the fluorescence assay for the binding affinity of sense and antisense peptides, a solution of a non-fluorescent peptide was gradually added to a solution of a fluorescent peptide, and the changes in fluorescence intensity were observed. Each sample was incubated for 5 min at room temperature prior to measurement to ensure equilibration and accurate readings. No further change in the fluorescence signal was observed with longer incubation times, thus ensuring that equilibrium had been reached.

Fluorescence spectra of the peptides and their complexes were measured at 25 °C in a quartz cuvette (*l* = 1 cm, cat. no. QS105.250, Hellma, Germany) using the OLIS RSM 1000F spectrofluorometer (OLIS Inc., Seattle, WA, USA), with an excitation wavelength of 280 nm, recording emission spectra between 310 and 416 nm for 30 s at 62 scans per second (a total of 1860 spectra were averaged for each titration point). Fluorescence (*F*) is given in fluorescence units (f. u.) calculated as the ratio of signals from the sample and reference photomultiplication tube. Each measurement was repeated three times, and the mean values were calculated. Data were analyzed using SPECFIT Global Analysis software (version 2.12) to model the binding interactions, considering concentration changes and wavelength-dependent fluorescence [56,57].

### 3.4. Docking Simulations for the Binding of Sense and Antisense Peptides or Antisense Peptides and Ammodytoxin A

The binding interactions between sense and antisense peptides as well as antisense peptides and AtxA were further evaluated using the HPEPDOCK server. This computational tool is designed for the blind docking of peptides to proteins that uses a hierarchical algorithm to predict the binding modes of protein–peptide complexes. Unlike methods that rely on extensive simulations to account for peptide flexibility, HPEPDOCK generates an ensemble of peptide conformations using its MODPEP program, enabling efficient and accurate docking predictions [48].

The scoring method used by the HPEPDOCK server integrates an iterative knowledge-based scoring function (ITScore-PP) developed for protein–protein docking [58]. The resulting docking scores are dimensionless and represent a relative ranking of the probability of binding positions rather than absolute binding energies. The scores prioritize the predicted binding poses but are not directly equivalent to binding constants or free energy changes and should be interpreted together with experimental validation to ensure accuracy and applicability.

The HPEPDOCK web server (http://huanglab.phys.hust.edu.cn/hpepdock/ (accessed on 15 October 2024)) was used with two inputs: the protein receptor structure in PDB format and the peptide sequence in FASTA format. The server output contains 100 docking models, each representing the lowest-energy structure of a cluster. The sequences in the AtxA structure corresponding to either S1 or S2 peptides were included as receptor binding residues in the advanced options of the web server interface.

In a comprehensive evaluation of fourteen docking programs for protein–peptide complexes, HPEPDOCK performed best in global docking for the entire benchmark dataset of 185 protein–peptide complexes [59]. The advantages of using HPEPDOCK include its specialized design for peptide–protein interactions and the consideration of peptide flexibility through an ensemble of up to 1000 initial peptide conformations. The choice of HPEPDOCK is in line with the objectives of this study as it provides a validated and robust platform for the investigation of potential binding interactions between peptides and proteins. In addition, the HPEPDOCK web server interface is straightforward and requires minimal user setup. The hybrid energy-based scoring method is expected to improve the accuracy of interface prediction and reduce false positives.

Understanding the molecular interactions between sense and antisense peptides is crucial for exploring the principles of molecular recognition and their implications for peptide design and drug discovery. Therefore, a detailed contacts analysis of the top-scoring docking models was performed to identify key interactions between the sense peptides and their antisense counterparts or AtxA using the Contacts and H-Bonds tools in ChimeraX (Figure S2, Supporting Information) [47].

The initial 3D structures of the S1, S1A1, and S1A2 pentapeptides were generated using the PEP-FOLD3 server (https://mobyle.rpbs.univ-paris-diderot.fr/cgi-bin/portal.py#forms::PEP-FOLD3 (accessed on 15 October 2024)), as HPEPDOCK was unable to generate initial structures from FASTA input. These predicted structures served as initial conformations for the docking simulations. The 3D structures of nonapeptides S2, S2A1, and S2A2 were successfully initiated by HPEPDOCK. PEP-FOLD3 is a computational tool developed for the de novo modeling of the 3D structures of linear peptides with a length of 5 to 50 amino acids. The modeling is based solely on sequence information and uses a Hidden Markov Model and fragment assembly to predict peptide conformations [60]. This combination enables fast and accurate prediction of peptide structure and typically provides results within minutes.

In tests performed with 56 peptides in aqueous solution, PEP-FOLD3 successfully generated conformations that matched experimentally determined structures for 80% of the target peptides. Furthermore, using a benchmark of 61 peptide–protein targets starting from the unbound form of the protein receptor, PEP-FOLD3 was able to generate peptide poses that deviated on average by 3.3 Å from the experimental conformation and provided a native-like pose for 52% of the targets in the first 10 clusters [60]. In addition to PEP-FOLD3, alternative AI-based approaches can also be used to generate initial peptide conformations. While PEP-FOLD3 was used for structure generation in this study, the use of alternative AI-based tools such as Boltz-1 [61] could provide additional structural diversity and improve docking predictions in future work.

## 4. Conclusions

This study combined fluorescence spectroscopy, experimental binding assays, and molecular docking simulations to evaluate the interactions between sense and antisense peptides designed to target the neurotoxic sites of AtxA. The results provide insight into the binding properties and potential applications of these peptides. The fluorescence measurements and in silico analyses revealed distinct physico-chemical properties among the studied peptides. In general, high fluorescence intensities of peptides are associated with negative GRAVY values, indicating their hydrophilic nature. Conversely, the low fluorescence intensities align with their higher aliphatic indices, which suggest less hydrophilic structures. These findings support the molecular recognition theory, suggesting complementary hydropathic properties between sense and antisense peptides.

The interactions between sense and antisense peptides were confirmed spectrofluorimetrically by measuring fluorescence intensity at various titrant concentrations. The calculated *K*_D_ values were in the micromolar range, indicating moderate to strong interactions. These experimental results align with the design principles of antisense peptides targeting neurotoxic regions. Overall, the lower *K*_D_ values observed for S1A2 and S2A1 compared to their counterparts indicate that specific amino acid substitutions can significantly enhance the binding affinity, providing valuable insights for optimizing peptide design for targeted interactions.

The experimental binding affinities generally align with the docking predictions in terms of relative trends, particularly for S1 interactions. However, the docking scores are not directly proportional to the measured *K*_D_ values, highlighting limitations in using docking simulations alone to predict absolute binding strengths. Experimental validation remains crucial to confirm binding strength and specificity. Together, these findings highlight the importance of integrating experimental and computational approaches to accurately evaluate peptide–protein interactions.

The confirmation of oligopeptide interactions, especially for peptides with a low number of amino acids, highlights their potential as therapeutic agents and warrants further investigation. The results support the development of antisense peptides as potential inhibitors of AtxA, with S1A2 and S2A1 emerging as promising candidates for therapeutic applications. Further studies are required for direct experimental validation of the binding of antisense peptides to the AtxA protein. This validation may be facilitated by the use of a recombinant wild-type AtxA, whose activity is comparable to that of the natural protein isolated from viper venom [62]. The use of recombinant AtxA could potentially enable controlled studies to evaluate the efficacy of antisense peptides as potential therapeutics in a high-throughput format.

While fluorescence intensity measurements were appropriate for the sense and antisense peptides in this study, employing additional techniques such as biolayer interferometry (BLI), surface plasmon resonance (SPR), or isothermal titration calorimetry (ITC) could provide more precise binding constant determinations and enhance the robustness of the findings. These methods also allow for greater flexibility in antisense peptide design, eliminating the constraint of requiring one peptide to fluoresce while the other does not. This broader approach could increase the likelihood of identifying antisense peptides with even higher binding affinities, advancing their potential therapeutic applications.

## Figures and Tables

**Table 1:** Sense amino acids and their complementary antisense amino acids [25]. Standard amino acid one-letter code is given in parentheses, and antisense amino acids are given in corresponding codes.

Sense Amino Acid	Antisense Amino Acid (Direction 5′-3′)	Antisense Amino Acid (Direction 3′-5′)
Isoleucine (I)	Y, N, D	Y
Valine (V)	H, N, D, Y	H, Q
Leucine (L)	E, K, Q	N, E, D
Phenylalanine (F)	K, E	K
Cysteine (C)	T, A	T
Methionine (M)	H	Y
Alanine (A)	R, G, S, C	R
Glycine (G)	P, S, T, A	P
Threonine (T)	G, S, C, R	C, W
Tryptophan (W)	P	T
Serine (S)	R, G, T, A	S, R
Tyrosine (Y)	I, V	M, I
Proline (P)	G, W, R	M, R
Histidine (H)	V, M	V
Aspartate (D)	I, V	L
Glutamate (E)	L, F	L
Asparagine (N)	I, V	L
Glutamine (Q)	L	V
Lysine (K)	F, L	F
Arginine (R)	A, S, P, T	A, S

**Table 2:** Physico-chemical properties of the sense and antisense peptides. The values of *λ*_max_ and *F* correspond to the spectra shown in Figure 3. Other values were obtained using the ExPASy ProtParam server (https://web.expasy.org/protparam/ (accessed on 12 June 2024)). The peptide labeling scheme is presented in Section 3.

Sequence	Label	*F* ^a^ /f. u.	*λ*_max_/nm	p*I*	Half-Life ^b^/h	II ^c^	AI ^d^	GRAVY ^e^
GMMILG	S1	0.80	345	5.52	30	35.63	130	1.883
YIYRNYPDF	S2	3.55	317	5.83	2.8	0.77	43.3	−1.078
SHHYEP	S1A1	2.37	320	5.93	1.9	101.1	0	−2.267
SHHYSP	S1A2	2.11	319	6.66	1.9	154.8	0	−1.817
INISIVGVK	S2A1	0.94	346	8.75	20	35.64	194.4	1.478
VNISVIGIK	S2A2	0.75	343	8.72	100	38.84	194.4	1.478

^a^ Fluorescence *F* is given in fluorescence units (f. u.) calculated as the ratio of signals from the sample and reference photomultiplication tube. ^b^ Estimated time it takes for half of the amount of protein in a cell to disappear after its synthesis in mammalian reticulocytes, in vitro [35]. ^c^ Estimate of the protein stability in vitro: a protein whose instability index (II) is smaller than 40 is predicted as stable, while a value above 40 predicts that the protein may be unstable [36]. ^d^ Aliphatic index (AI) of a protein is defined as the relative volume occupied by aliphatic side chains (alanine, valine, isoleucine, and leucine). It may be regarded as a positive factor for the increase in thermostability of globular proteins [37]. ^e^ Grand average of hydropathy values based on the Kyte–Doolittle scale [27]. Positive GRAVY values indicate hydrophobic, whereas negative values indicate hydrophilic.

**Table 3:** Calculated values of dissociation constants *K*_D_ for the studied sense and antisense peptide interactions. Standard deviations of the *K*_D_ values are given in parentheses. Peptide labeling scheme is presented in Section 3.

Sense peptide	Antisense Peptide	*K*_D_ (*σ*)/μM
S1	S1A1	4.9 (0.7)
	S1A2	1.4 (0.5)
S2	S2A1	1.1 (0.1)
	S2A2	8.9 (0.8)

**Table 4:** Specification of the synthesized peptides provided by the manufacturer (GenScript Biotech B.V., Rijswijk, The Netherlands; lot no. U648UEK070-1).

Peptide Type	Sequence	Label *	*M*/g mol^−1^	Purity/%
Sense	GMMILG	S1	621	95.9
	YIYRNYPDF	S2	1250	98.9
Antisense	SHHYEP	S1A1	769	98.5
	SHHYSP	S1A2	727	99.7
	INISIVGVK	S2A1	942	95.3
	VNISVIGIK	S2A2	942	95.5

* S denotes sense peptide and A denotes antisense peptide. For each sense peptide (Sx), two corresponding antisense peptides (SxAy) were investigated, where x and y values can each be either 1 or 2.

## Data Availability

The original data presented in the study are openly available in the Zenodo repository at https://doi.org/10.5281/zenodo.14524531. This repository contains all experimental and computational data associated with the study of sense–antisense peptide interactions targeting neurotoxic sites in ammodytoxin A (AtxA), including the following: Peptide Specifications: Detailed information on the synthesized peptides used in the study (GenScript Biotech B.V., Rijswijk, The Netherlands). Protparam Output: Computational analysis of peptide physico-chemical properties generated using the Expasy ProtParam tool. SPECFIT Data: Fluorescence titration results analyzed using SPECFIT software (version 2.12), including association constants, spectral fits, and residual plots. HPEPDOCK Outputs: Docking models and scores for the interaction of sense and antisense peptides or antisense peptides with AtxA (PDB: 3G8G). Initial 3D structures of the S1, S1A1 and S1A2 pentapeptides were generated using the PEP-FOLD3 server. ChimeraX Session Files: Molecular visualization sessions highlighting docking results, binding interfaces, and peptide conformations. Fluorescence Spectra: Experimental fluorescence spectra of stock solutions of sense and antisense peptides.

## Supplementary Materials

The following supporting information can be downloaded at: https://www.mdpi.com/article/10.3390/molecules30040903/s1; Figure S1: Results of the singular value decomposition (SVD) analysis of fluorescence data for titrations involving antisense peptides S1A1, S1A2, S2A1, and S2A2 (Scree plots); Figure S2: Settings for the Contacts and H-bonds tools in ChimeraX used for the detailed analysis of interactions between sense and antisense peptides; Figure S3. Global docking simulations of the sense peptides S1 (GMMILG) and S2 (YIYRNYPDF) with their respective antisense peptides.; Table S1: Summary of the major interactions for each antisense peptide with its sense counterpart, obtained using the Contacts and H-bonds tools in ChimeraX; Table S2: Summary of the major interactions for each antisense peptide with AtxA, obtained using the Contacts and H-bonds tools in ChimeraX.
