# Supplementary material for: Design and Binding Affinity of Antisense Peptides for Snake Venom Neutralization
Source: Molecules. 2025 Feb 15;30(4):903. doi: 10.3390/molecules30040903 (PMC11858715; doi:10.3390/molecules30040903)
Supplement: Supplementary file 1 [file molecules-30-00903-s001.zip › molecules-3429172-supplementary.pdf]

# Design and Binding Affinity of Antisense Peptides for Snake Venom Neutralization

**Ivan Biruš, Tino Šeba, Marin Marić, Mario Gabričević and Tin Weitner ***
Department of General and Inorganic Chemistry, University of Zagreb Faculty of Pharmacy and Biochemistry, A. Kovačića 1, 10000 Zagreb, Croatia

* **Correspondence:** tin.weitner@pharma.unizg.hr

## Supporting information

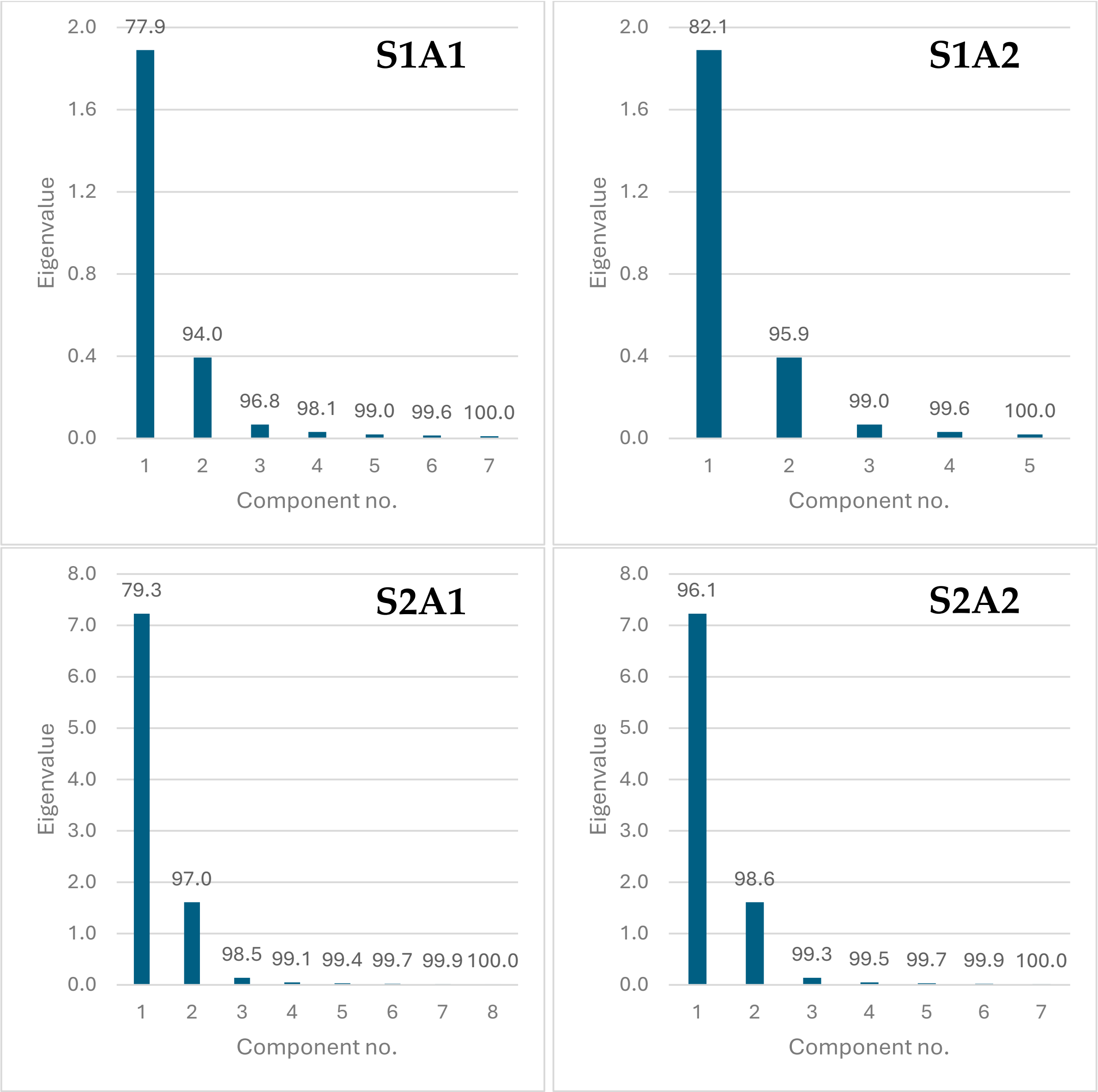


**Figure S1.** Results of the singular value decomposition (SVD) analysis of fluorescence data for titrations involving antisense peptides S1A1, S1A2, S2A1, and S2A2 (Scree plots; https://doi.org/10.1198/106186007X256080). The x-axis represents the component number, and the y-axis shows the square sum of the corresponding singular values. Data labels above each bar display the cumulative percentage of the total variance accounted for by the components. The analysis highlights that the first two components capture the majority of the signal variance, with diminishing contributions from higher-order components.

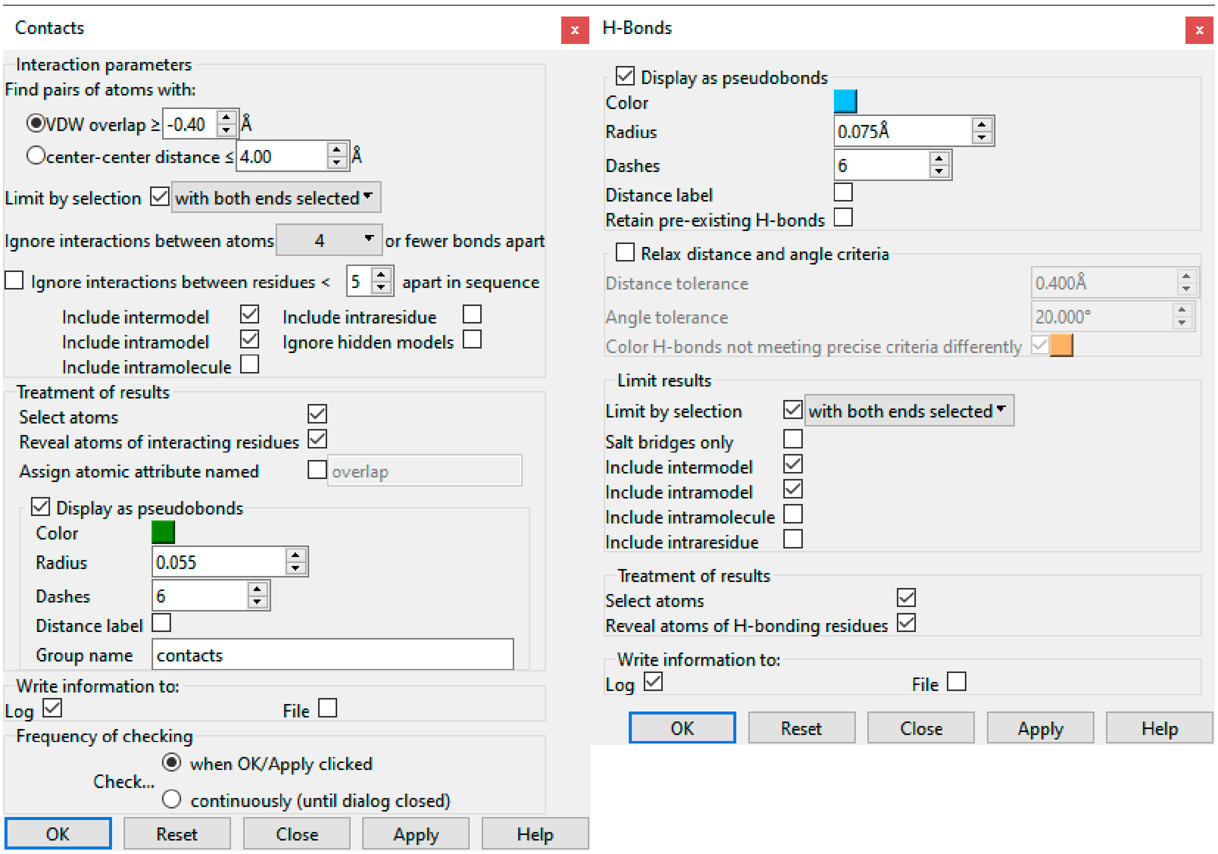


**Figure S2.** Settings for the Contacts and H-bonds tools in ChimeraX used for the detailed analysis of interactions between sense and antisense peptides. The Contacts tool (https://www.cgl.ucsf.edu/chimerax/docs/user/commands/clashes.html) parameters were configured to identify pairs of atoms with van der Waals (VDW) overlap ≥ -0.40 Å or center-center distances ≤ 4.00 Å, ignoring interactions between atoms separated by 4 or fewer bonds. The H-bonds tool (https://www.cgl.ucsf.edu/chimerax/docs/user/tools/hbonds.html) was configured to detect hydrogen bonds based on strict distance (≤ 0.400 Å) and angle (≤ 20°) tolerances.

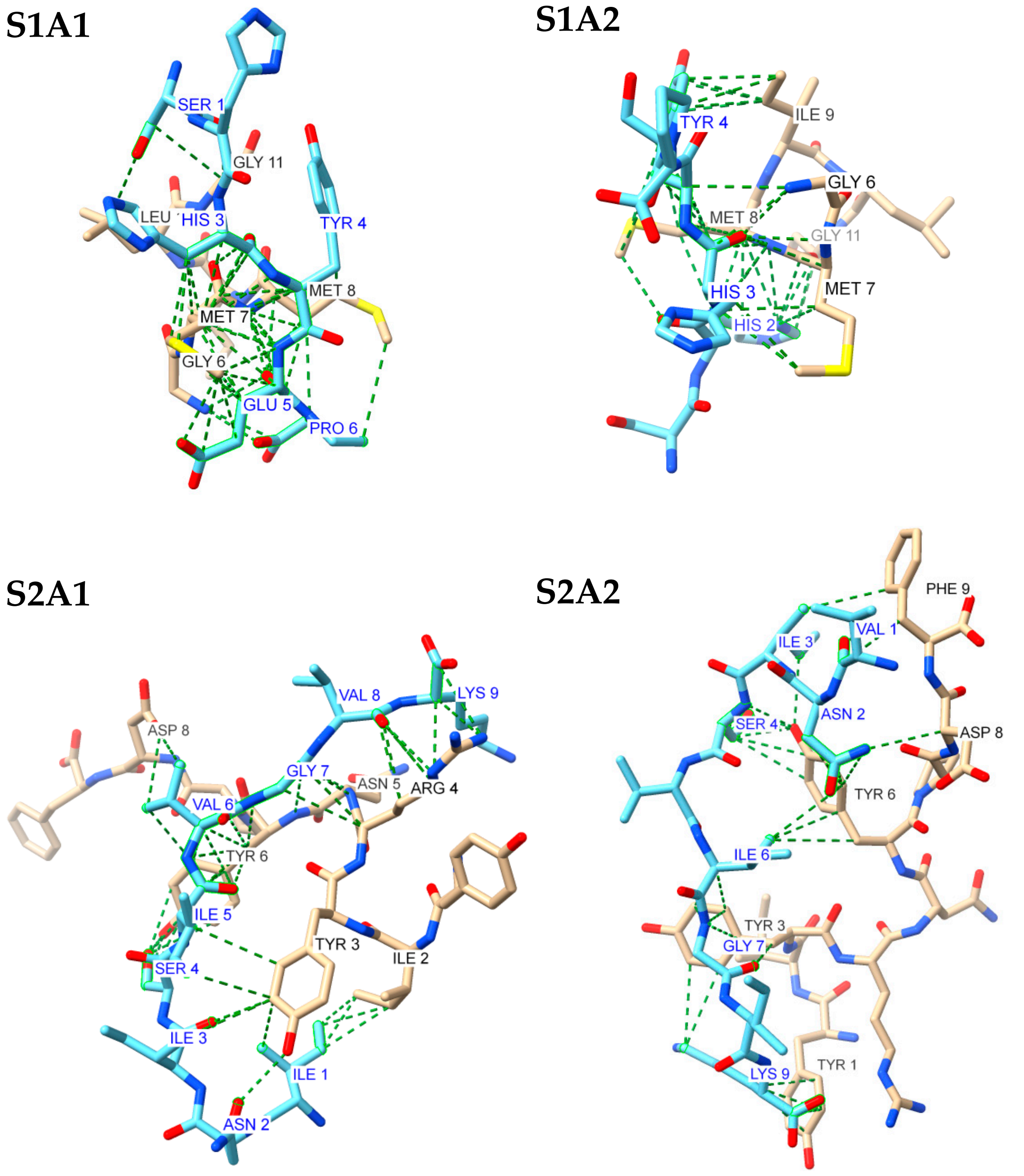


**Figure S3.** Global docking simulations of the sense peptides S1 (GMMILG) and S2 (YIYRNYPDF) (tan models) with their respective antisense peptides (blue models). Docking simulations were performed using the HPEPDOCK web server, while visualization and contact analysis were carried out in UCSF ChimeraX (version 1.8). Interacting residues involved in the interface are labeled in black for the sense peptides and blue for the antisense peptides. Interactions are highlighted as green dashed pseudobonds, with atom colors distinguishing key elements: oxygen (red), nitrogen (blue), and sulfur (yellow).

**Table S1.** Summary of the major interactions for each antisense peptide with its sense counterpart, obtained using the Contacts and H-bonds tools in ChimeraX (https://doi.org/ 10.1002/pro.3943).

| Peptide | Residue Pair | Interaction Type |
|---|---|---|
| S1A1 | MET 2 - GLU 5 | Hydrophobic interactions |
| | MET 2 - HYS 3 | Hydrophobic interactions |
| | MET 3 - TYR 4 | Potential sulfur-π interaction |
| | MET 3 - GLU 5 | Potential H-bond* |
| S1A2 | MET 2/3 - HIS 2/3 | Hydrophobic interaction |
| | ILE 4 - TYR4 | Hydrophobic interaction; potential π-π stacking** |
| | MET 3 - TYR 4 | Potential sulfur-π interaction |
| | MET 3 - HIS 3 | Potential H-bond* |
| S2A1 | ILE 2 - ILE 1 | Hydrophobic interaction |
| | TYR 6 - SER 4 | Hydrophobic interaction |
| | TYR 6 - VAL 6 | Hydrophobic interaction |
| | TYR 3 - ILE 3 | Hydrophobic interaction; potential π-π stacking** |
| S2A2 | TYR 6 - SER 4 | Hydrophobic interaction |
| | LYS 9 - TYR 1 | Hydrophobic interaction |
| | TYR 6 - ASN 2 | Hydrophobic interaction; potential electrostatic interaction |
| | LYS 9 - TYR 1 | Hydrophobic interaction |

* Hydrogen bonds can be inferred based on geometric criteria involving only the donor and acceptor atoms. In such cases, potential hydrogen bonds can be inferred by evaluating distances and angles between these heavy atoms, even in the absence of explicit hydrogen atoms. This approach allows for the identification of probable hydrogen bonds, but they should be interpreted with caution, considering the limitations of not modeling hydrogen atoms explicitly.

** Recent research suggests that π-π interactions involving non-aromatic groups in protein structures may have been underappreciated in computational models. Unlike classical π-π stacking between two aromatic rings, these interactions involve planar $sp^2$-hybridized orbital systems, including backbone amides and the side chains of residues such as Asn, Gln, Glu, and Asp (https://doi.org/10.7554/eLife.31486).

**Table S2.** Summary of the major interactions for each antisense peptide with AtxA, obtained using the Contacts and H-bonds tools in ChimeraX (https://doi.org/ 10.1002/pro.3943).

| Peptide | Residue Pair | Interaction Type |
|---|---|---|
| S1A1 | TYR 21 - HIS 3 | $\pi$-$\pi$ stacking, potential H-bond* |
| | VAL 30 - GLU 5 | Hydrophobic, potential electrostatic |
| | ASP 48 - TYR 4 | $\pi$-$\pi$ stacking, potential H-bond |
| | CYS 44 - TYR 4 | Potential sulfur-$\pi$,** potential H-bond |
| S1A2 | PHE 5 - TYR 4 | $\pi$-$\pi$ stacking |
| | ILE 9 - TYR 4 | Hydrophobic |
| | GLY 6 - HIS 2 | Potential H-bond |
| | PHE 96 - TYR 4 | $\pi$-$\pi$ stacking |
| | CYS 44 - TYR 4 | Sulfur-$\pi$ |
| S2A1 | ARG 108 - ILE 1 | Hydrogen bond |
| | LYS 37 - VAL 8 | Hydrophobic |
| | TYR 110 - VAL 8 | Hydrophobic |
| | ASN 109 - ASN 2 | Potential H-bond |
| S2A2 | LYS 9 - TYR 110 | Hydrogen bond |
| | TYR 105 - VAL 1 | Hydrophobic |
| | LEU 114 - ILE 8 | Hydrophobic |
| | ARG 108 - ILE 3 | Potential H-bond |

* While traditionally hydrogen bonding is associated with electronegative atoms such as nitrogen, oxygen, and fluorine, studies have shown that carbon atoms can also engage in hydrogen bonding interactions. Under certain circumstances, methyl groups and other $sp^3$ hybridized carbons can form weak CH···O interactions and thus contribute to the stability and dynamics of protein structures (https://doi.org/10.1002/prot.24724; https://doi.org/DOI: 10.1021/acs.jcim.2c00015).

** Sulfur-$\pi$ interactions are a type of non-covalent interaction that occurs between the sulfur atom of sulfur-containing amino acids (such as cysteine and methionine) and the aromatic rings of amino acids like tyrosine. Sulfur is one of the most polarizable atoms in proteins, which allows it to engage effectively with the electron cloud of the aromatic ring (https://doi.org/10.1071/CH14598).
